# Platelet-Derived Microparticles From Obese Individuals: Characterization of Number, Size, Proteomics, and Crosstalk With Cancer and Endothelial Cells

**DOI:** 10.3389/fphar.2019.00007

**Published:** 2019-01-22

**Authors:** Rosalia Grande, Melania Dovizio, Simone Marcone, Paulina B. Szklanna, Annalisa Bruno, H. Alexander Ebhardt, Hilary Cassidy, Fionnuala Ní Áinle, Anna Caprodossi, Paola Lanuti, Marco Marchisio, Geltrude Mingrone, Patricia B. Maguire, Paola Patrignani

**Affiliations:** ^1^Department of Neurosciences, Imaging and Clinical Sciences, Università degli Studi “G. d’Annunzio”, Chieti, Italy; ^2^Center of Research on Aging and Translational Medicine (CeSI-MeT), Università degli Studi “G. d’Annunzio”, Chieti, Italy; ^3^Systems Biology Ireland, Conway SPHERE Research Group Ireland, University College Dublin, Dublin, Ireland; ^4^Conway SPHERE Research Group Ireland, UCD Conway Institute, University College Dublin, Dublin, Ireland; ^5^Department of Internal Medicine, Catholic University, Rome, Italy; ^6^Department of Medicine and Aging Sciences, Università degli Studi “G. d’Annunzio”, Chieti, Italy

**Keywords:** microparticles, platelets, obesity, proteomics, cellular cross-talk

## Abstract

**Rationale:** Obesity is a risk factor for atherothrombosis and various cancers. However, the mechanisms are not yet completely clarified.

**Objectives:** We aimed to verify whether the microparticles (MPs) released from thrombin-activated platelets differed in obese and non-obese women for number, size, and proteomics cargo and the capacity to modulate *in vitro* the expression of (i) genes related to the epithelial to mesenchymal transition (EMT) and the endothelial to mesenchymal transition (EndMT), and (ii) cyclooxygenase (COX)-2 involved in the production of angiogenic and inflammatory mediators.

**Methods and Results:** MPs were obtained from thrombin activated platelets of four obese and their matched non-obese women. MPs were analyzed by cytofluorimeter and protein content by liquid chromatography-mass spectrometry. MPs from obese women were not different in number but showed increased heterogeneity in size. In obese individuals, MPs containing mitochondria (mitoMPs) expressed lower CD41 levels and increased phosphatidylserine associated with enhanced Factor V representing a signature of a prothrombotic state. Proteomics analysis identified 44 proteins downregulated and three upregulated in MPs obtained from obese vs. non-obese women. A reduction in the proteins of the α-granular membrane and those involved in mitophagy and antioxidant defenses-granular membrane was detected in the MPs of obese individuals. MPs released from platelets of obese individuals were more prone to induce the expression of marker genes of EMT and EndMT when incubated with human colorectal cancer cells (HT29) and human cardiac microvascular endothelial cells (HCMEC), respectively. A protein, highly enhanced in obese MPs, was the pro-platelet basic protein with pro-inflammatory and tumorigenic actions. Exclusively MPs from obese women induced COX-2 in HCMEC.

**Conclusion:** Platelet-derived MPs of obese women showed higher heterogeneity in size and contained different levels of proteins relevant to thrombosis and tumorigenesis. MPs from obese individuals presented enhanced capacity to cause changes in the expression of EMT and EndMT marker genes and to induce COX-2. These effects might contribute to the increased risk for the development of thrombosis and multiple malignancies in obesity.

**Clinical Trial Registration:** www.ClinicalTrials.gov, identifier NCT01581801.

## Introduction

The activation of platelets in response to tissue damage is an early event in the reparative process (Gawaz et al., 2005). However, in some circumstances, the cascade of biological processes involved in tissue healing can be affected, thus translating into the development of a chronic inflammatory state which promotes the development and progression of numerous disorders, including atherothrombosis and colorectal cancer (CRC) (Gawaz et al., 2005; Patrignani and Patrono, 2016). Since platelets up-take proteins and genetic material from plasma (Best et al., 2015), the platelet phenotype is influenced by the individual clinical condition.

Platelets release small membrane-bound microparticles (MPs) containing bioactive proteins and genetic material which can be delivered to recipient cells, including immune, endothelial, epithelial and cancer cells (Dovizio et al., 2018); through this mechanism cells acquire novel phenotypes and functions which may promote the development of pathological states (Dovizio et al., 2018).

Lifestyle factors, such as western style dietary habits, and lack of physical activities associated with overweight and obesity, are risk factors for various types of cancer (Basen-Engquist and Chang, 2011). Excess body fat is potentially a modifiable cancer risk factor (Basen-Engquist and Chang, 2011). However, the biological mechanisms underlying the relationship between obesity and cancer have not been completely elucidated yet. We hypothesize that platelet-derived MPs and their proteomic content are altered in obesity, thus promoting cancer.

This study aimed to characterize the number, size, and proteome of MPs generated *in vitro* in response to thrombin from platelets of obese women and their matched lean controls. Moreover, we performed experiments *in vitro* to explore the capacity of platelet-derived MPs of both groups to influence the expression of marker genes of epithelial- and endothelial-mesenchymal transition (EMT and EndMT, respectively), in the HT29 human colorectal adenocarcinoma cells and human cardiac microvascular endothelial cells (HCMEC). The effect of MPs of both groups on endothelial cyclooxygenase (COX)-2 expression, a pro-angiogenic and inflammatory pathway (Wang and DuBois, 2004), was also evaluated.

## Materials and Methods

### Subjects

We studied four obese and four non-obese women. Demographic and clinical characteristics of the two groups are reported in Table 1. All individuals were enrolled at the Unit of Obesity disorders, Policlinico Gemelli, Catholic University of Rome (Italy). Obesity was defined as a BMI (Body Mass Index; calculated as weight in kilograms divided by the square of height in meters) of 30 and above. The two groups had comparable age (43.50 ± 5.33 and 43.25 ± 4.35 years, mean ±*SD*, respectively) and did not present hypertension, diabetes mellitus or dyslipidemia (Table 1). They did not use any medication. The two groups differed for the BMI (49.50 ± 1.12 and 21.89 ± 1.01, respectively, *P* < 0.01) (Table 1). The experimental protocol was approved by the Ethics Committee of Policlinico Gemelli (Catholic University, Rome, Italy) (Clinicaltrials.gov Registration number NCT01581801). This study was carried out following the recommendations of the Declaration of Helsinki and the approved guidelines from the Ethics Committee of Policlinico Gemelli. After signing the informed consent, all individuals underwent blood collection.

**Table 1:** Demographic and clinical characteristics of healthy and obese individuals.

	Healthy subjects	Obese individuals	*P*-values^a^
Number	4	4	
Sex, female (%)	4 (100%)	4 (100%)	
Age (years)	43.25 ± 4.35	43.50 ± 5.33	>0.05
BMI (kg/m^2^)	21.89 ± 1.01	49.50 ± 1.12	<0.0001
Diabetes, n (%)	0 (0.00)	0 (0.00)	
Hypertension, n (%)	0 (0.00)	0 (0.00)	
Epatic steatosis, n (%)	0 (0.00)	0 (0.00)	
Total cholesterol	184.00 ± 10.35	175.80 ± 8.34	>0.05
HDL mg/dL	64.25 ± 1.03	50.00 ± 5.80	>0.05
LDL mg/dL	106.30 ± 8.08	100.00 ± 11.32	>0.05
Glucose mg/dL	85.75 ± 2.394	95.25 ± 3.794	>0.05
Drugs, n (%)	0 (0.00)	0 (0.00)	

*Data are expressed as mean ±SD. ^a^By Fisher’s Exact Test or Unpaired Student’s t-test, as appropriate.*

### Platelet Microparticle (MP) Preparation

Washed platelets were obtained, as previously described (Dovizio et al., 2013; Vasina et al., 2013), and analyzed for the contamination of leukocytes [identified for their positivity to Syto16 fluorescent nucleic acid stain (Thermo Fisher Scientific, Milan, Italy) and CD45 (using mAb from BD Biosciences, Milan, Italy)] and erythrocytes [recognized for the surface expression of CD235a (using mAB from BD Biosciences)] by flow cytometry. Platelets were stimulated with thrombin (1 U/ml, Sigma-Aldrich) for 30 min at 37°C to generate MPs, as previously described (Vasina et al., 2013). Platelet MPs were characterized using a flow cytometer with mAb against CD41 (CD41-PerCP-Cy5.5, BD Biosciences) and the presence of whole platelets in the suspension was ruled out. Platelets and MPs were analyzed by FacsVerse cytometer (BD Biosciences), and data were examined using FACSuite v 1.0.5 (BD Biosciences) software.

### Flow Cytometry Analysis of Platelet MPs

After resuspension of MP pellet in Annexin buffer (BD Biosciences), platelet MPs were labeled with MitoStatus-APC (Thermo-Fisher)/Phalloidin (Sigma-Aldrich, Milan, Italy)/CD41-PerCP-Cy5.5 (BD Biosciences)/AnnexV-V500 (BD Biosciences), as reported in the manufacturer’s instructions, and counted by flow cytometry. MPs were gated based on their size, and the scatter properties were analyzed by running Megamix Plus beads (Biocytex, Marseille, France) at the same photomultiplier (PMT) voltages used for MP detection. Phalloidin negative events (of total MPs or MitoStatus positive MPs) were analyzed for CD41 expression. CD41+ events were then evaluated for their positivity to AnnexinV.

### Assessment of MP Protein Content by Liquid Chromatography-Mass Spectrometry (LC-MS/MS)

Samples were prepared as previously described (Parsons et al., 2018). Protein concentration was assessed by Bradford protein assay (Bio-Rad, Hercules, CA, United States) using bovine serum albumin (BSA) (Sigma-Aldrich) as standard for the calibration curve and for each sample 30 μg proteins were precipitated with 95% acetone (4:1 acetone: sample volume) overnight. LC-MS/MS analysis of proteins was performed, as previously reported (Parsons et al., 2018). Briefly, dried protein pellets were resuspended in 8 M Urea/ 25 mM Tris–HCl, pH 8.2, at 37°C with gentle agitation. Disulfide bonds were reduced with 5 mM DTT and protected with 15 mM iodoacetamide. Proteins were first digested with Lys-C (1:100; Promega, Madison, WI, United States) followed by digestion with trypsin (1:100; Promega). Peptides were purified using ZipTipC18 pipette tips according to manufacturer instructions (Millipore, Billerica, MA, United States) and resuspended in 1% formic acid. Approximately 2 μg of purified peptides were injected per LC-MS/MS analysis using an Ultimate3000 nano-LC system coupled to a hybrid quadrupole-orbitrap mass spectrometer (Q Exactive, Thermo Fisher Scientific). Peptides were separated by an increasing acetonitrile gradient from 2 to 33 % in a linear LC gradient of 40 min on a C18 reverse phase chromatography column packed with 2.4 μm particle size, 300 Å pore size C18 material (Dr. Maisch GmbH, Ammerbuch-Entringen, Germany) to a length of 120 mm in a column with a 75 μm ID, using a flow rate of 250 nL/min. All data were acquired with the mass spectrometer operating in an automatic data-dependent acquisition mode (DDA, shotgun). A full MS service scan at a resolution of 70,000, AGC target 3e6 and a range of *m/z* 350–1600 was followed by up to 12 subsequent MS/MS scan with a resolution of 17,500, AGC target 2e4, isolation window *m/z* 1.6 and a first fix mass of *m/z* 100. Dynamic exclusion was set to 40 s.

The MS data have been submitted to the PRIDE proteomics identification database ^1^ under accession numbers PXD011563. Downstream analysis of proteomic data was performed by Perseus software (version 1.6.0.7). LFQ intensities of three technical replicates were averaged, and only the proteins present in at least 50% of the samples in one group (healthy donors and obese subjects) were considered identified. Proteins found to be differentially expressed between groups (*P*-value <0.05, FDR 0.01) were subjected to pathway mapping analysis and were distributed into categories according to their cellular component, molecular function, and biological process using Ingenuity Pathway Analysis (IPA) [QIAGEN (Redwood City, CA)] or STRING Database (Version 10.5). The molecular activation prediction (MAP) algorithm in IPA was used to predict the upstream and downstream effects of activation and inhibition of associated network functions. STRING ^2^ was also used to generate protein-protein interaction networks, and the KEGG pathway enrichment analysis tool in PANTHER classification system was also applied to these networks. Finally, the STRING was used to validate IPA findings and provide unique perspectives based on each tool.

### Effects of Platelet-Derived MPs on the Expression of Target Genes in Cancer and Endothelial Cells

The human colon carcinoma cell line HT29 and human cardiac microvascular endothelial cells (HCMEC) were purchased by ATCC (Milan, Italy) and Lonza (Milan, Italy), respectively, and cultured following the manufactory’s instructions. HT29 or HCMEC cells (0.25 × 10^6^) were incubated for 24 h with MPs (0.25 × 10^8^) generated from thrombin activated platelets of obese and non-obese individuals. MPs were assessed for the capacity to cause changes in the expression of marker genes of EMT and EndMT when incubated with HT29 cells and HCMEC, respectively (Dovizio et al., 2013). Finally, MPs were assessed for the capacity to induce endothelial COX-2 expression (Dovizio et al., 2013). mRNA levels were evaluated by qPCR as previously described (Dovizio et al., 2013).

### Statistical Analysis

All data are reported as mean ± *SD* unless otherwise stated. Statistical analysis was performed by using GraphPad Prism Software (version 5.00 for Windows; GraphPad, San Diego, CA, United States). Student’s *t-*test was used to compare the means of two independent groups to each other; instead, one-way analysis of variance followed by Newman-Keuls post-test was used to compare the means of more than two independent groups. Thus, were considered statistically significant *P*-values <0.05.

## Results

Washed platelets were isolated from the whole blood (Dovizio et al., 2013) of four obese individuals and as many non-obese controls. The cellular suspension contained predominantly platelets [98.24 ± 1.10% (mean ± SD)]. MPs, released from washed platelets activated with thrombin (1 IU/mL), were collected. The total platelet-derived MP count per μL, assessed by cytofluorimeter, was not significantly different in non-obese and obese individuals (19608 ± 9236 vs. 24259 ± 5796, respectively).

**FIGURE 1:**
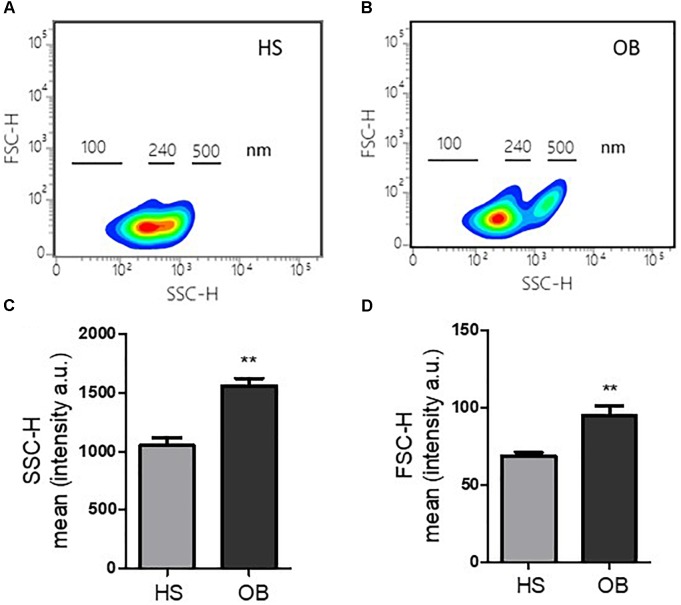
Features of MPs released from platelets of obese and non-obese women. **(A,B)** Density plots of forward scatter height (FSC-H) vs. side scatter height (SSC-H) of a typical MP suspension from non-obese (HS) and obese (OB) individuals, and size distribution. **(C,D)** Fluorescence intensity of FSC-H and SSC-H parameters reported as arbitrary unit (a.u.) (*n* = 4 for each group); ^∗∗^*P* < 0.01 vs. HS.

We studied the biophysical light scatter properties of MPs obtained in the two groups using cytofluorimeter. The density plot of side scatter (SSC) vs. forward scatter (FSC) of a typical MP suspension from non-obese and obese individuals (Figures 1A,B, respectively) showed a different size distribution between the two groups. Interestingly, in the obese individuals, MPs with size >240 nm were detected (Figures 1A,B). Platelet MPs from obese individuals had a significantly higher SSC and FSC signal intensity than non-obese individuals (Figures 1C,D, respectively).

We characterized the proteomic profile of MPs generated from thrombin activated platelets of non-obese and obese individuals. Thus, proteins from MPs were digested and analyzed by LC-MS/MS. In total, we identified 214 proteins in MPs. In Supplementary Table 1, the list of proteins identified in MPs is reported. Statistical analysis identified 47 proteins significantly modulated between the two groups (44 were downregulated while three were upregulated in MPs of obese vs. non-obese) (Supplementary Table 2). A further three proteins were detected only in MPs released from thrombin-stimulated platelets of obese individuals [ubiquitin like modifier activating enzyme 1 (UBA1), glutathione reductase, mitochondrial (GSR) and tyrosine-protein kinase (CSK)], while two proteins were present only in the MPs from non-obese individuals [calnexin (CANX) and cGMP-specific 3,5-cyclic phosphodiesterase (PDE5A)] (Supplementary Table 2).

**FIGURE 2:**
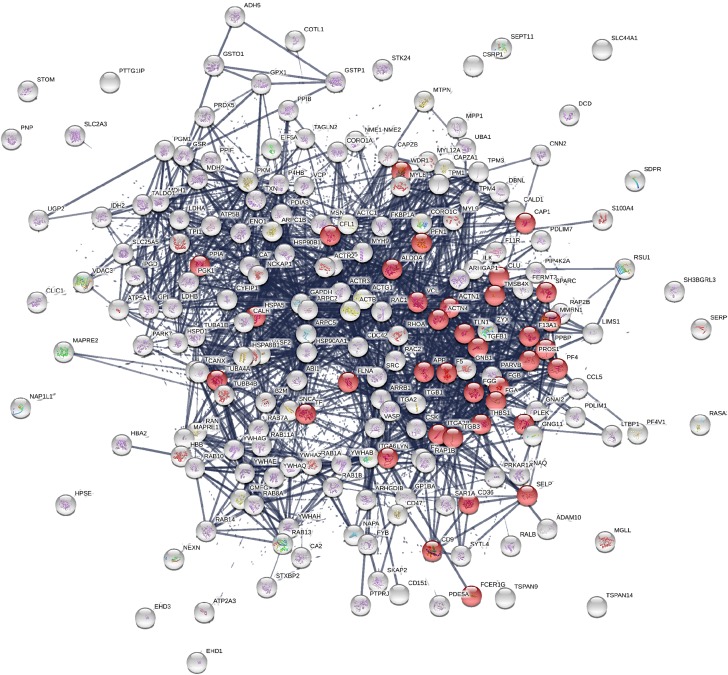
Protein-Protein interaction network of the 214 identified proteins: network nodes represent proteins, network edges indicate the strength of data support (STRING v10.5). Proteins associated with “Platelet activation” pathway are highlighted in red.

**FIGURE 3:**
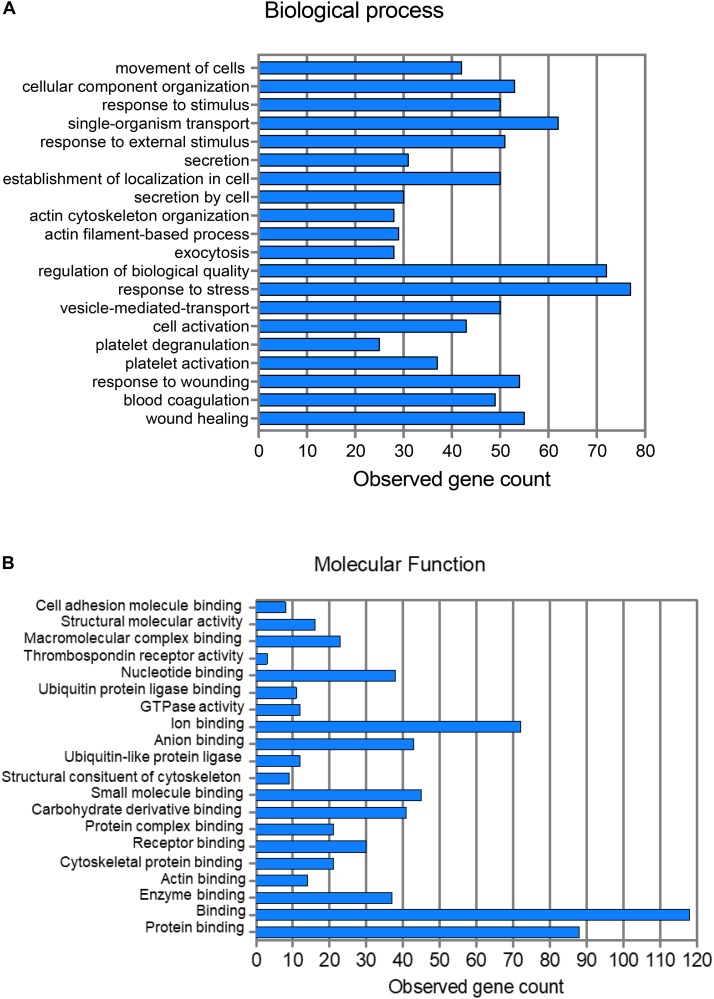
Pathway analysis of 214 proteins showing the top 20 biological processes **(A)** and molecular functions **(B)**.

A network analysis of all proteins was determined using the STRING database (Figure 2). Biological process and molecular function terms associated with the MP proteins are reported in Figures 3A,B, respectively. Many biological processes were associated with platelet activation and degranulation (Figure 3A).

**FIGURE 4:**
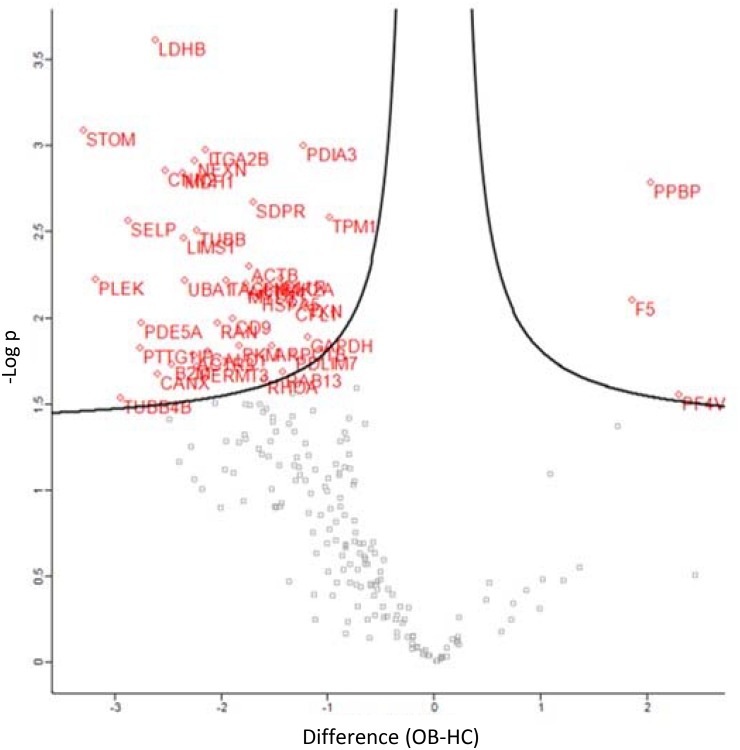
Volcano plot displaying the 47 differential expressed proteins between obese (OB) and healthy control (HC) platelet-derived MPs; the *y-axis* corresponds to the mean expression value of log10 (*p*-value), and the *x-axis* displays the difference values (OB-HC), the red dots represent the differentially expressed proteins (*P* < 0.05), and the gray dots represent the proteins whose expression levels did not reach statistical significance (*P* > 0.05).

**FIGURE 5:**
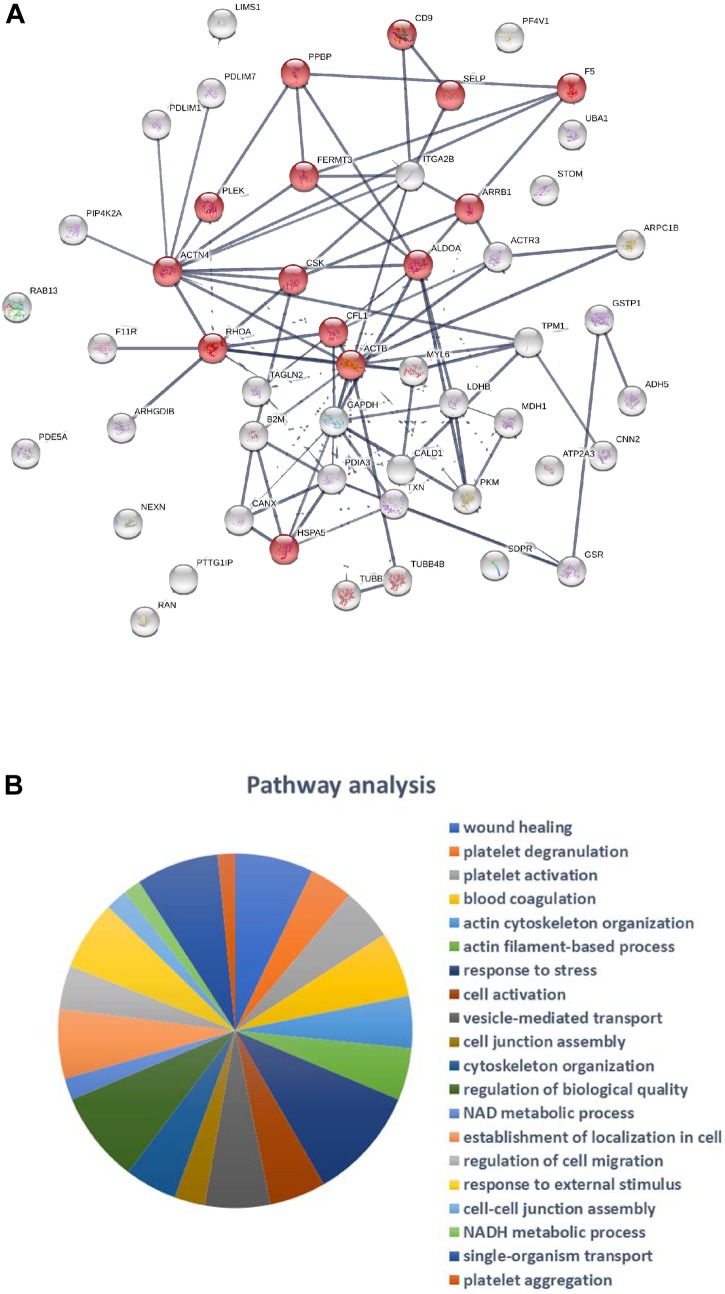
**(A)** STRING network representation of the 47 platelet-derived MP proteins significantly modulated between non-obese and obese individuals: network nodes represent proteins; network edges indicate the strength of data support; significantly modulated proteins associated with “Platelet activation” pathway are highlighted in red. **(B)** The 20 top-ranked categories (false discovery rate) of KEGG pathways significantly enriched in our dataset.

Classification of the 47 modulated proteins was performed by STRING database and KEGG pathway enrichment analysis. The results showed that the proteins mapped to platelet functions, such as platelet activation and degranulation, and blood coagulation, but also to the regulation of cell migration, wound healing and vesicle-mediated transport (Figures 4, 5A,B). Moreover, 20 top-ranked categories of KEGG pathways significantly enriched in our dataset were associated with different platelet functions (Figure 5B).

**FIGURE 6:**
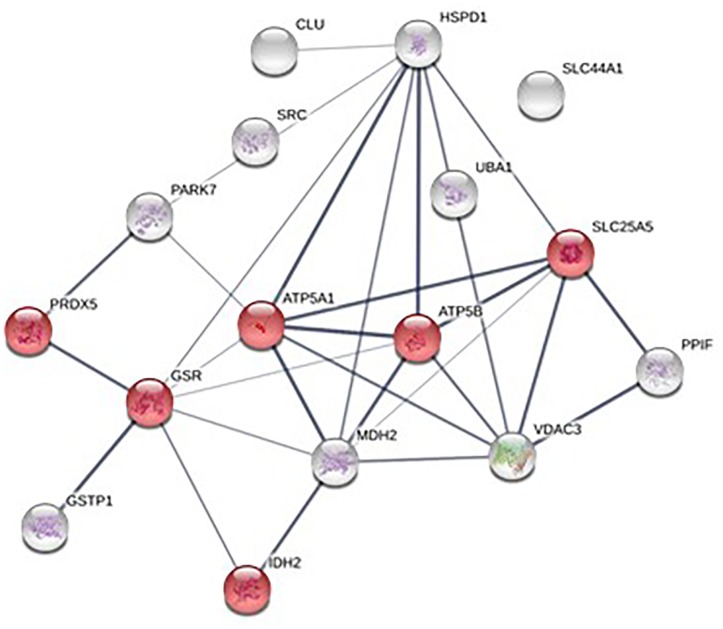
Protein-protein interaction network of the 16 mitochondrial proteins identified in platelet-derived MPs; mitochondrial proteins associated with “Oxidation-reduction processes” are highlighted in red.

**FIGURE 7:**
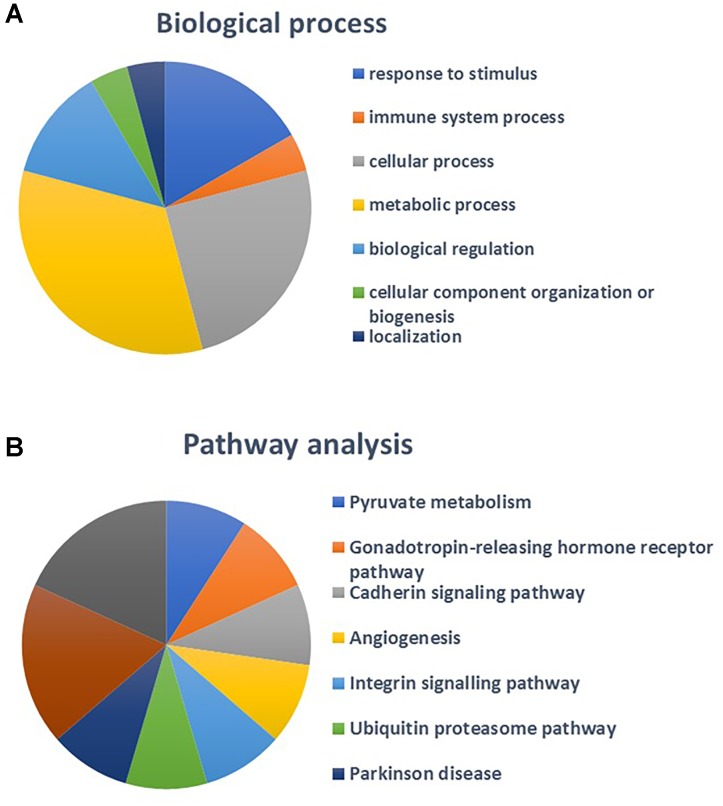
Biological processes **(A)** and pathway analysis **(B)** of identified mitochondrial proteins.

The number of MPs positive for mitochondria (mitoMPs) generated from thrombin-activated platelets was comparable in both groups (4384 ± 1497and 5867 ± 4441 number/μL, respectively). They represented the 23.30 ± 16.41 and 25.38 ± 8.08 %, respectively, of total MP population. Interestingly, 16 mitochondrial proteins were identified in our proteomic analysis of platelet-derived MPs (Supplementary Table 1). Pathway analysis performed using STRING database showed that the identified mitochondrial proteins mapped to regulation of integrin signaling pathway, angiogenesis, and pyruvate metabolism (Figures 6, 7A,B). Among the mitochondrial proteins, two were detected only in obese MPs (UBA1, GSR) whereas one was downregulated in MPs from obese vs. non-obese individuals (glutathione S-transferase pi 1, GSTP1) (Supplementary Table 2).

It is noteworthy the fact that two major glycolytic enzymes were among the 44 reduced proteins, lactate dehydrogenase B (LDHB, a subunit of lactate dehydrogenase enzyme; *P* = 0.00024; fold-change, 0.162) and pyruvate kinase muscle isozyme (PKM) (*P* = 0.0145; fold-change, 0.281) (Supplementary Table 2).

In obese MPs, reduced levels of P-selectin (gene name, SELP) (*P* = 0.003, fold-change, 0.136) and CD41 (i.e., integrin subunit alpha 2b; gene name ITGA2B) (*P* = 0.001; fold-change, 0.225) were found (Supplementary Table 2).

**FIGURE 8:**
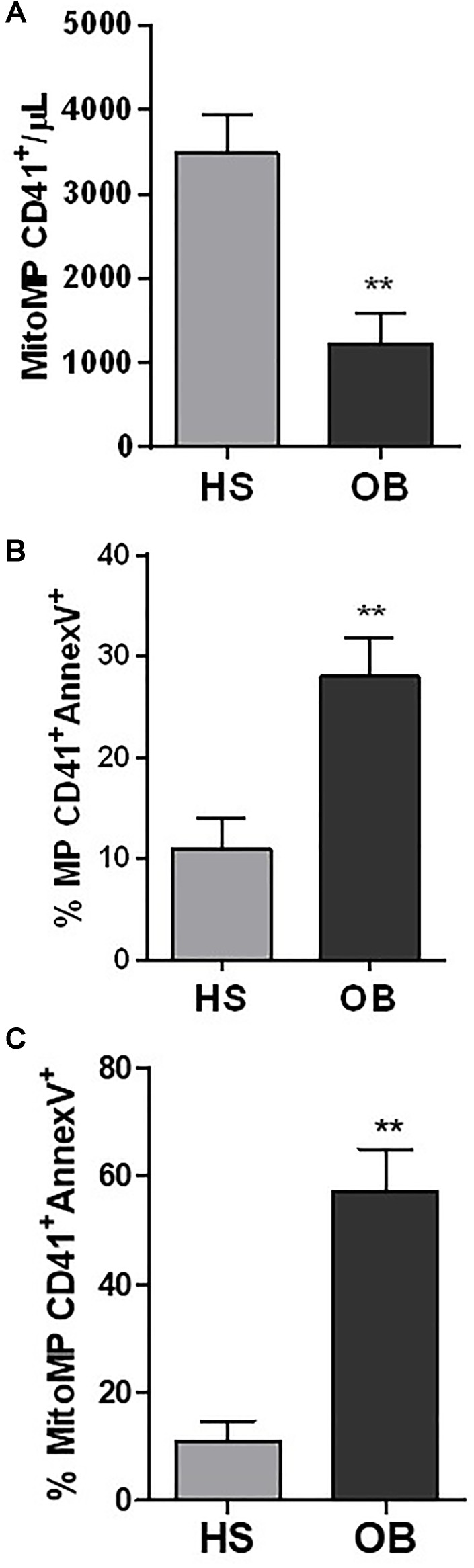
Characterization of platelet-derived MPs isolated from healthy (HS) and obese (OB) individuals by flow-cytometry. **(A)** The count of platelet mitoMP CD41^+^ was reported as the number of mitoMP/μL. **(B)** The % of MP CD41^+^/Annexin V^+^ and **(C)** mitoMP CD41^+^/Annexin V^+^ are reported. ^∗∗^*P* < 0.01 vs. HS (*n* = 4 for each group).

Using flow cytometry, the number of mitoMP CD41^+^ were lower in obese (1231 ± 727.9 number/μL) vs. non-obese individuals (3486 ± 1021 number/μL) (Figure 8A) (*P* < 0.01).

It is known that platelet-derived MPs can expose phosphatidylserine (PS) which in turn binds annexin V and that the annexin V-PS bond represents a true reflection of MP procoagulant activity (Connor et al., 2009). In non-obese and obese individuals, % of total MP CD41^+^ which binds annexin V was 10.95 ± 6.73 vs. 27.97 ± 7.54%, respectively (*P* < 0.01) (Figure 8B). MitoMP CD41^+^annexinV^+^ were 10.88 ± 9.56 and 56.99 ± 16.01%, respectively (*P* < 0.01) (Figure 8C). These results are consistent with the proteomic data showing that Factor V was upregulated in obese MPs vs. non-obese MPs (*P* = 0.008, fold-change, 3.635) (Supplementary Table 2).

**FIGURE 9:**
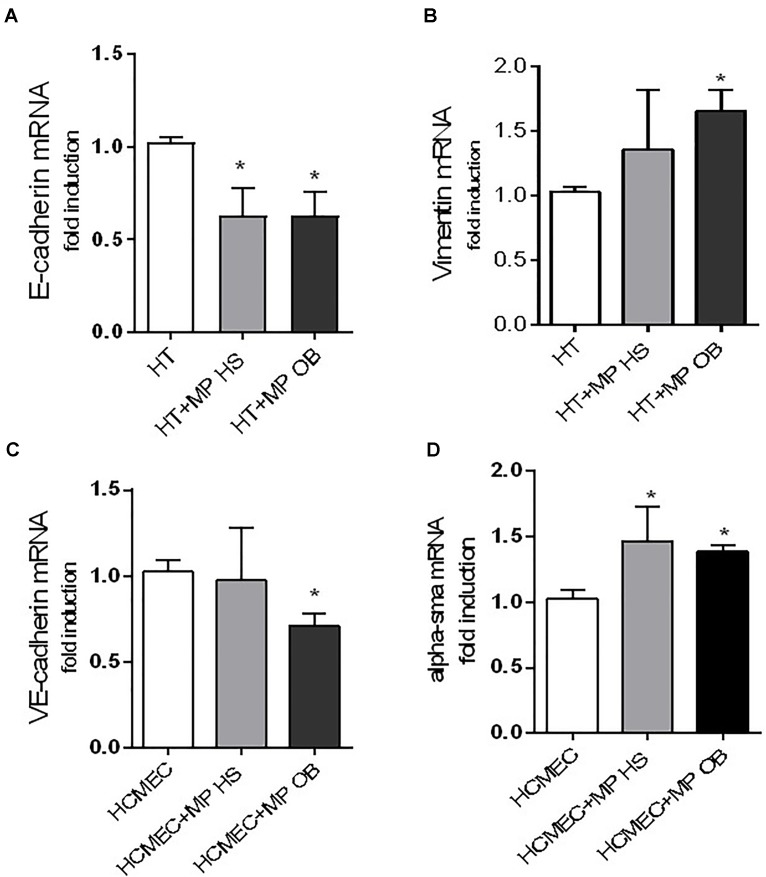
Effects of MPs on the expression of gene markers of EMT and EndMT. Co-culture experiments between colon adenocarcinoma cells HT29 (0.25 × 10^6^) **(A,B)** or human coronary microvascular endothelial cells (HCMEC) (0.25 × 10^6^) **(C,D)** and MPs (0.25 × 10^8^) from obese (OB) and healthy (HS) individuals for 24 h were reported. Gene expression was evaluated by qPCR and normalized to those of GAPDH as control and expressed as fold-change. Data are reported as mean ± SEM (*n* = 4, for each experimental condition); ^∗^*P* < 0.05 vs. HT29 cultured alone or HCMEC cultured alone.

Also, we characterized the property of MPs generated from obese and non-obese thrombin-activated platelets for their property to alter the expression of molecular markers involved in EMT, a key process mediating the progression of malignant tumors (Kalluri and Weinberg, 2009). As shown in Figure 9A, platelet MPs of both groups incubated with HT29 cells caused a significant downregulation of expression levels of E-cadherin, a typical epithelial marker. This effect was associated with an increase in the mesenchymal marker vimentin, which was significant only with MPs isolated from obese platelets (Figure 9B).

Microparticles of both groups were studied for the capacity to alter the expression profile of marker genes of EndMT in HCMEC. Only MPs obtained from obese individuals caused a significant downregulation of VE-cadherin in HCMEC (a typical endothelial marker) (Figure 9C). In contrast, α – SMA was significantly upregulated by MPs derived from both groups (Figure 9D).

**FIGURE 10:**
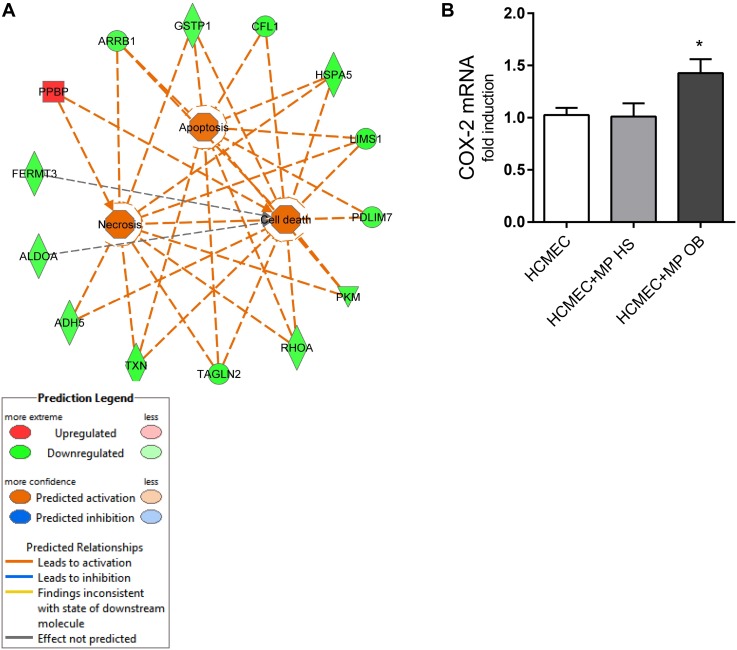
**(A)** Pathway analysis of modulated MP proteins obtained with IPA for “apoptosis and cell death” signaling is reported. IPA analysis showed regulatory relationships between down-regulated (green) and upregulated (red) proteins; the Molecular Activation Prediction tool showed that “apoptosis, cell death, and necrosis” are positively regulated in obese subjects (orange lines); gray line indicates that the effect is not predicted. **(B)** In co-culture experiment of HCMEC (0.25 × 10^6^) and platelet MPs from OB and HS individuals (0.25 × 10^8^) for 24 h, the gene expression of COX-2 was evaluated by qPCR, normalized to GAPDH levels as control, and expressed as fold-change. Data are reported as mean ± SEM (*n* = 4 for each experimental condition); ^∗^*P* < 0.05 vs. HCMEC cultured alone.

IPA analysis of the 47 modulated proteins showed that some processes enriched in our proteomic analysis might regulate apoptosis and cell death signaling (Figure 10A).

Finally, we assessed the effect of MPs to induce the pro-inflammatory and pro-angiogenic gene COX-2 (Wang and DuBois, 2004) in endothelial cells (Figure 10B). Platelet MPs from obese individuals induced COX-2 while MPs from non-obese did not.

## Discussion

In the present study, we aimed to verify whether obesity influences the number, size and proteome of MPs generated *in vitro* from platelets in response to thrombin. Moreover, we studied the capacity of platelet-derived MPs to influence the expression of marker genes of EMT and EndMT and COX-2 *in vitro*.

We found that MPs, released from activated platelets of obese individuals, were not different in number as compared with non-obese controls, but were characterized by greater heterogeneity in size distribution. However, in obese women, the count of mitoMPs positive for CD41 was significantly lower. This finding can be explained by the fact that in obesity a strong platelet activation associated with enhanced oxidative stress occurs (Santilli et al., 2011). These events may lead to the alteration of mitochondrial functions and proteolytic cleavage of proteins.

Proteomics data showed reduced levels of pyruvate kinase (PKM) in obese MPs, which is a regulator of mitophagy (i.e., the process of the removal of damaged mitochondria) via enhanced pyruvate formation (Park et al., 2015). A defect in platelet mitophagy response has been described in diabetes (Lee et al., 2016) and may lead to increased thrombosis in response to oxidative stress. The mitochondrial protein, GSTP1 was also reduced in obese MPs vs. non-obese MPs. This protein plays an important role in antioxidant defenses (Meiers, 2010).

Platelet MPs obtained from obese individuals had reduced levels of the α-granular transmembrane (TM) proteins P-selectin (SELP) and stomatin (gene name, STOM). Two other TM proteins found on α-granule membranes had decreased expression on obese MPs, i.e., alpha-IIb (gene name, ITGA2B) and CD9 antigen (gene name, CD9). These changes may reflect an alteration in membrane fluidity in obesity leading to a biological modification in platelet and MP membranes (Tangorra et al., 1988; Cazzola et al., 2011). Interestingly, another transmembrane protein the junctional adhesion molecule A (gene name, F11R), which functions as an endogenous inhibitor of platelet function (Naik et al., 2011), was also reduced in obese MPs.

Microparticles from platelets of obese individuals presented three proteins that were not detectable in non-obese MPs. Among them, there is UBA1 which catalyzes the first step in ubiquitin conjugation to mark cellular proteins for degradation through the ubiquitin-proteasome system (Ciechanover and Schwartz, 1998). Its presence in obese MPs might play a role in the reduced levels of many proteins detected vs. MPs of non-obese individuals.

Soluble megakaryocyte-derived α-granule components were increased in obese MPs, including coagulation Factor V (gene name, F5) and pro-platelet basic protein (gene name, PPBP). Factor V functions as a membrane-bound cofactor and plays an essential role in hemostasis through its profound influence on the production of thrombin (Camire, 2010). Enhanced content of Factor V, together with increased exposure of membrane PS in the MP of obese individuals, may account for the pro-thrombotic risk associated with obesity. Interestingly, in MPs of obese individuals, high levels of PPBP (also known as CXCL7) were found. PPBP is the precursor of platelet basic protein (PBP), a platelet-derived growth factor stored in platelet α-granules and is a potent chemoattractant and activator of neutrophils. CXCL7 mediates different effects through its G-protein-coupled receptors CXCR-1 and CXCR-2, which activate the ERK and PI3 kinase pathways (Grépin et al., 2014). The activation of these receptors expressed in HT29 cells (Desurmont et al., 2015) and endothelial cells (Grépin et al., 2014) might contribute to EMT and EndMT and cellular migration.

Another protein highly upregulated in MPs from obese individuals is a variant form of platelet factor 4 (PF4 variant 1/CXCL4L1, gene name, PF4V1) (Supplementary Table 2). It is a potent inhibitor of angiogenesis (Sarabi et al., 2011) and may induce random endothelial cell migration (Sarabi et al., 2011) thus possibly contributing to EndMT.

Platelet-derived MPs from obese, but not from non-obese, individuals, induced COX-2 expression in HCMEC. This effect might play a role in the obesity promotion of cancer, since COX-2-dependent prostaglandin (PG) E_2_ induces angiogenesis at the earliest stage of tumor development (Wang and DuBois, 2004).

The limitations of this study are: (i) the small number of individuals analyzed; (ii) only women were studied; (iii) the effect of MPs on the expression of EMT marker genes was evaluated on HT29 cell line, not on primary cancer cells derived from patients; (iv) the impact of MPs on functional assays of EMT and EndMT was not studied.

The strength of this study is the development of a proteomics approach to determine the composition of MPs generated from activated platelets. Also, this study provides data on the variability of MP generation from activated platelets, such as number and size, in obese and non-obese individuals. This information will be helpful to design larger clinical studies, in this setting.

In conclusion, our results suggest that and the assessment of proteomics signature of MPs, generated from thrombin-activated platelets, can be suitable for monitoring the efficacy of lifestyle, pharmacologic, and surgical options in obesity. However, larger studies should be performed to validate our findings.

## Author Contributions

PP, PBM, and GM conceptualized and designed the study. RG, MD, SM, PBS, HE, HC, PL, MM, and AC performed the data acquisition, analysis, or interpretation of data. PP, PBM, MD, and RG drafted the manuscript. AB and FN critically revised the manuscript for important intellectual content. All authors provided approval for publication of the content, and agreed to be accountable for all aspects of the work in ensuring that questions related to the accuracy or integrity of any part of the work are appropriately investigated and resolved.

## Conflict of Interest Statement

The authors declare that the research was conducted in the absence of any commercial or financial relationships that could be construed as a potential conflict of interest.

## Footnotes

1 www.ebi.ac.uk/pride

2 www.string-db.org
