# Supplementary material for: Platelet-Derived Microparticles From Obese Individuals: Characterization of Number, Size, Proteomics, and Crosstalk With Cancer and Endothelial Cells
Source: Front Pharmacol. 2019 Jan 22;10:7. doi: 10.3389/fphar.2019.00007 (PMC6349702; doi:10.3389/fphar.2019.00007)
Supplement: Supplementary file 1 [file Table_1.pdf]

Supplementary Table 1

Gene names	Protein names	Protein IDs	Sequence coverage [%]	Unique sequence coverage [%]	Peptides	Unique peptides	Mol. weight [kDa]	Q-value	Score	Intensity	MS/MS count	Coefficient of variation
PDLIM1	PDZ and LIM domain protein 1	O00151	42,9	42,9	8	8	36,071	0	185,28	3511800000	146	0,428
CLIC1	Chloride intracellular channel protein 1	O00299	73	73	13	13	26,922	0	300,19	6547100000	189	0,124
ADAM10	Disintegrin and metalloproteinase domain-containing protein 10	O14672	11,2	11,2	4	4	84,141	0	162,05	2014800000	97	0,229
MYL12A	Myosin regulatory light chain 12A	P19105	8,2	8,2	1	1	19,794	0	52,977	814230000	26	1,904
FYB	FYN-binding protein	O15117	7	7	5	5	85,386	0	56,945	1097500000	62	1,259
ARPC1B	Actin-related protein 2/3 complex subunit 1B	O15143	18,8	18,8	5	5	40,949	0	150,74	3377900000	78	0,201
ARPC2	Actin-related protein 2/3 complex subunit 2	O15144	33,3	33,3	7	7	34,333	0	96,045	1774300000	58	1,233
ARPC5	Actin-related protein 2/3 complex subunit 5	O15511	54,3	54,3	6	6	16,32	0	77,484	2309100000	73	1,412
ACTN4	Alpha-actinin-4	O43707	23,7	12,5	17	6	104,85	0	54,064	322720000	15	1,690
GMFG	Glia maturation factor gamma	O60234	37,3	37,3	4	4	16,801	0	103,65	550950000	28	2,151
WDR1	WD repeat-containing protein 1	O75083	62,9	62,9	26	26	66,193	0	323,31	10614000000	334	0,035
SKAP2	Src kinase-associated phosphoprotein 2	O75563	17,3	17,3	6	6	41,216	0	94,998	624240000	35	1,852
TSPAN9	Tetraspanin-9	O75954	7,5	7,5	2	2	26,779	0	38,621	425780000	18	1,201
PDE5A	cGMP-specific 3,5-cyclic phosphodiesterase	O76074	5,7	5,7	4	4	99,984	0	27,096	126600000	9	2,138
SDPR	Serum deprivation-response protein	O95810	21,4	21,4	7	7	47,173	0	178,24	2307900000	84	0,248
LDHA	L-lactate dehydrogenase A chain	P00338	30,1	30,1	11	11	36,688	0	323,31	1653700000	97	0,649
*GSR	Glutathione reductase, mitochondrial	P00390	5	5	2	2	56,256	0	17,8	102070000	13	1,852
F13A1	Coagulation factor XIII A chain	P00488	21,3	21,3	15	15	83,266	0	323,31	5950500000	216	0,185
PNP	Purine nucleoside phosphorylase	P00491	30,4	30,4	6	6	32,118	0	135,77	1120800000	55	0,868
PGK1	Phosphoglycerate kinase 1	P00558	72,2	72,2	23	23	44,614	0	323,31	5151000000	248	0,021
F2	Prothrombin	P00734	8	8	3	3	70,036	0	45,972	556110000	36	1,852
CA2	Carbonic anhydrase 2	P00918	34,6	34,6	7	7	29,246	0	72,913	848600000	63	1,381
TGFB1	Transforming growth factor beta-1	P01137	31	31	7	7	44,341	0	71,843	909980000	38	1,852
IGKC	Ig kappa chain C region	P01834	34	34	2	2	11,609	0	60,156	339090000	25	1,472
FGA	Fibrinogen alpha chain	P02671	7,9	7,9	5	5	94,972	0	51,211	634480000	45	1,852
FGB	Fibrinogen beta chain	P02675	12,2	12,2	4	4	55,928	0	34,953	314800000	16	1,852
FGG	Fibrinogen gamma chain	P02679	24,1	24,1	7	7	51,511	0	311,49	818430000	50	1,382
PPBP	Platelet basic protein	P02775	56,2	56,2	12	12	13,894	0	323,31	72157000000	448	0,062
PF4	Platelet factor 4	P02776	43,6	11,9	5	2	10,845	0	57,616	21706000000	56	1,852
TF	Serotransferrin	P02787	5	5	3	3	77,063	0	108,99	139820000	12	2,148
CAT	Catalase	P04040	18,8	18,8	5	5	59,755	0	52,326	337300000	14	1,459
ALDOA	Fructose-bisphosphate aldolase A	P04075	62,1	62,1	14	14	39,42	0	323,31	7766100000	177	0,267
GAPDH	Glyceraldehyde-3-phosphate dehydrogenase	P04406	56,4	56,4	16	16	36,053	0	323,31	20923000000	366	0,011
GNAI2	Guanine nucleotide-binding protein G(i) subunit alpha-2	P04899	24,2	24,2	9	9	40,45	0	108,28	1044900000	48	1,412
APP	Amyloid beta A4 protein	P05067	4,9	4,9	2	2	86,942	0	34,867	142270000	16	2,828
ITGB3	Integrin beta-3	P05106	28,3	28,3	20	20	87,057	0	323,31	27803000000	340	0,028
*SLC25A5	ADP/ATP translocase 2	P05141	16,4	9,7	4	2	32,852	0	26,122	808900000	40	1,852
ITGB1	Integrin beta-1	P05556	22,7	22,7	13	13	88,414	0	209,36	2450500000	97	0,326

*ATP5B	ATP synthase subunit beta, mitochondrial	P06576	10,2	10,2	4	4	56,559	0	37,112	962670000	31	1,852
ENO1	Alpha-enolase	P06733	85	78,8	26	24	47,168	0	323,31	11224000000	340	0,019
GPI	Glucose-6-phosphate isomerase	P06744	29,4	29,4	11	11	63,146	0	163,74	1291600000	55	0,733
TPM3	Tropomyosin alpha-3 chain	P06753	24,6	8,4	5	2	32,95	0	14,849	375150000	25	1,852
LDHB	L-lactate dehydrogenase B chain	P07195	33,5	33,5	10	10	36,638	0	141,63	2045000000	122	0,246
GPX1	Glutathione peroxidase 1	P07203	21,7	21,7	3	3	22,088	0	23,196	226950000	16	1,852
PROS1	Vitamin K-dependent protein S	P07225	8,1	8,1	3	3	75,122	0	28,226	182590000	11	1,852
P4HB	Protein disulfide-isomerase	P07237	10,6	10,6	4	4	57,116	0	49,233	409580000	33	1,852
GP1BA	Platelet glycoprotein Ib alpha chain	P07359	12,3	12,3	6	6	71,539	0	98,934	1092700000	59	1,143
TUBB	Tubulin beta chain	P07437	58,3	17,3	14	4	49,67	0	323,31	6295600000	204	0,131
PFN1	Profilin-1	P07737	99,3	99,3	21	21	15,054	0	323,31	27216000000	335	0,019
HSP90AA1	Heat shock protein HSP 90-alpha	P07900	11,3	9,7	7	6	84,659	0	112,5	685100000	34	1,852
LYN	Tyrosine-protein kinase Lyn	P07948	27,9	27,9	9	8	58,573	0	79,908	618520000	35	1,915
THBS1	Thrombospondin-1	P07996	21,4	21,4	22	22	129,38	0	323,31	5551300000	227	0,331
ITGA2B	Integrin alpha-IIb	P08514	22,1	22,1	16	16	113,38	0	323,31	22523000000	383	0,017
PLEK	Pleckstrin	P08567	51,4	51,4	17	17	40,124	0	323,31	6328200000	154	0,484
*GSTP1	Glutathione S-transferase P	P09211	52,4	52,4	6	6	23,356	0	140,07	1940000000	88	0,430
SPARC	SPARC	P09486	23,4	23,4	4	4	34,632	0	126,85	2532500000	71	1,381
TPM1	Tropomyosin alpha-1 chain	P09493	21,5	6	4	1	32,708	0	94,637	677850000	42	1,852
HSPA1B	Heat shock 70 kDa protein 1B	P0DMV9	31,7	21,1	11	7	70,051	0	89,157	665860000	46	1,043
TXN	Thioredoxin	P10599	30,5	30,5	2	2	11,737	0	27,016	434100000	23	1,901
PRKAR1A	cAMP-dependent protein kinase type I-alpha regulatory subunit	P10644	22,8	22,8	5	5	42,981	0	80,874	721550000	27	1,206
PF4V1	Platelet factor 4 variant	P10720	56,7	26	6	3	11,553	0	159,47	43347000000	171	0,224
*HSPD1	60 kDa heat shock protein, mitochondrial	P10809	11,2	11,2	5	5	61,054	0	38,955	485680000	37	1,852
*CLU	Clusterin	P10909	14,9	14,9	5	5	52,494	0	51,557	588810000	29	1,852
HSPA5	78 kDa glucose-regulated protein	P11021	25,1	22,6	12	11	72,332	0	197,81	1796600000	115	0,170
HSPA8	Heat shock cognate 71 kDa protein	P11142	45,4	38,7	21	18	70,897	0	323,31	9665300000	294	0,024
SLC2A3	Solute carrier family 2, facilitated glucose transporter member 3	P11169	21,8	21,8	6	6	53,924	0	143,58	1103600000	50	1,112
RALB	Ras-related protein Ral-B	P11234	24,8	24,8	4	4	23,408	0	31,154	454400000	19	1,896
ADH5	Alcohol dehydrogenase class-3	P11766	6,7	6,7	1	1	39,724	0	51,525	194510000	10	1,993
F5	Coagulation factor V	P12259	3,1	3,1	5	5	251,7	0	96,358	1403400000	81	0,936
ACTN1	Alpha-actinin-1	P12814	54,9	45,6	38	27	103,06	0	323,31	20919000000	507	0,019
*SRC	Proto-oncogene tyrosine-protein kinase Src	P12931	22,6	22,6	8	7	59,834	0	93,863	850500000	41	1,200
CCL5	C-C motif chemokine 5	P13501	40,7	40,7	5	5	9,9896	0	49,369	3398300000	59	1,852
PKM	Pyruvate kinase PKM	P14618	36,3	36,3	16	16	57,936	0	323,31	4856900000	176	0,171
HSP90B1	Endoplasmin	P14625	19,2	19,2	10	10	92,468	0	101,07	1161600000	53	1,548
RAC2	Ras-related C3 botulinum toxin substrate 2	P15153	50	31,2	8	5	21,429	0	57,846	1216400000	35	1,852
SELP	P-selectin	P16109	24,2	24,2	14	14	90,833	0	282,11	3127500000	141	0,041
PECAM1	Platelet endothelial cell adhesion molecule	P16284	32,2	32,2	16	16	82,535	0	323,31	4123400000	173	0,132
CD36	Platelet glycoprotein 4	P16671	21,4	21,4	9	9	53,053	0	255,93	3492500000	144	0,884
ITGA2	Integrin alpha-2	P17301	9,6	9,6	8	8	129,29	0	66,787	472350000	45	1,852
VCL	Vinculin	P18206	32,9	32,9	28	28	123,8	0	323,31	11313000000	279	0,336
CSRP1	Cysteine and glycine-rich protein 1	P21291	49,2	49,2	7	7	20,567	0	133,6	1308800000	52	0,426
FLNA	Filamin-A	P21333	39,9	39,9	82	82	280,74	0	323,31	64097000000	1162	0,021
CD9	CD9 antigen	P21926	26,8	26,8	9	9	25,416	0	323,31	10802000000	155	0,131

*UBA1	Ubiquitin-like modifier-activating enzyme 1	P22314	3,7	3,7	2	2	117,85	0	12,536	83741000	10	2,140
NME2	Nucleoside diphosphate kinase B	P22392	24,3	24,3	3	3	17,298	0	17,86	160020000	12	1,852
ITGA6	Integrin alpha-6	P23229	22,7	22,7	19	19	126,6	0	253,88	3061100000	118	0,449
PPIB	Peptidyl-prolyl cis-trans isomerase B	P23284	52,3	52,3	8	8	23,742	0	67,819	1382700000	62	1,148
CFL1	Cofilin-1	P23528	75,9	68,7	20	17	18,502	0	323,31	29829000000	449	0,020
MYL9	Myosin regulatory light polypeptide 9	P24844	8,1	8,1	2	2	19,827	0	50,297	375660000	26	1,906
*ATP5A1	ATP synthase subunit alpha, mitochondrial	P25705	20,6	20,6	8	8	59,75	0	188,37	928910000	57	1,852
MSN	Moesin	P26038	32,2	32,2	17	17	67,819	0	323,31	3374700000	159	0,246
S100A4	Protein S100-A4	P26447	33,7	33,7	4	4	11,728	0	86,299	2056700000	71	1,201
STOM	Erythrocyte band 7 integral membrane protein	P27105	48,6	48,6	10	10	31,73	0	323,31	13275000000	177	0,041
YWHAQ	14-3-3 protein theta	P27348	20,8	20,8	3	3	27,764	0	45,917	524190000	22	1,427
CALR	Calreticulin	P27797	26,4	26,4	7	7	48,141	0	54,253	487370000	26	1,852
CANX	Calnexin	P27824	16,9	16,9	4	4	67,567	0	29,614	275360000	6	1,472
*PRDX5	Peroxiredoxin-5, mitochondrial	P30044	39,3	39,3	4	4	22,086	0	86,248	528580000	28	2,828
PDIA3	Protein disulfide-isomerase A3	P30101	41,2	41,2	13	13	56,782	0	201,77	3119000000	131	0,287
FCER1G	High affinity immunoglobulin epsilon receptor subunit gamma	P30273	23,3	23,3	1	1	9,6674	0	17,64	337760000	20	1,897
*PPIF	Peptidyl-prolyl cis-trans isomerase F, mitochondrial	P30405	39,1	39,1	6	6	22,04	0	60,092	567640000	36	1,557
SERPINB1	Leukocyte elastase inhibitor	P30740	37,2	37,2	8	8	42,741	0	159,45	1357600000	71	0,710
CORO1A	Coronin-1A	P31146	23	21,3	8	7	51,026	0	74,602	1384500000	51	1,852
YWHAB	14-3-3 protein beta/alpha	P31946	35	23,2	6	3	28,082	0	94,606	705070000	16	1,852
MYH9	Myosin-9	P35579	41,3	41,3	69	69	226,53	0	323,31	16397000000	691	0,033
PGM1	Phosphoglucomutase-1	P36871	9,3	9,3	3	3	61,448	0	39,293	223720000	18	2,828
TAGLN2	Transgelin-2	P37802	48,7	48,7	7	7	22,391	0	116,2	1182700000	60	0,682
TALDO1	Transaldolase	P37837	18,4	18,4	5	5	37,54	0	54,294	251310000	12	1,988
SNCA	Alpha-synuclein	P37840	26,4	26,4	4	4	14,46	0	38,563	221350000	14	1,901
MDH1	Malate dehydrogenase, cytoplasmic	P40925	15,9	15,9	3	3	36,426	0	43,987	296820000	17	1,893
*MDH2	Malate dehydrogenase, mitochondrial	P40926	22,8	22,8	4	4	35,503	0	30,317	397060000	27	1,296
CSK	Tyrosine-protein kinase CSK	P41240	5,3	5,3	2	2	50,704	0,002874	11,04	80199000	6	1,852
CAPZB	F-actin-capping protein subunit beta	P47756	34,3	34,3	6	6	31,35	0	126,42	654440000	19	1,178
LIMS1	LIM and senescent cell antigen-like-containing domain protein 1	P48059	72,3	72,3	21	21	37,251	0	323,31	6846200000	210	0,267
PIP4K2A	Phosphatidylinositol 5-phosphate 4-kinase type-2 alpha	P48426	5,2	5,2	2	2	46,224	0	19,288	161360000	7	1,852
CD151	CD151 antigen	P48509	6,7	6,7	1	1	28,295	0	35,426	520950000	30	1,852
CCT5	T-complex protein 1 subunit epsilon	P48643	13,1	13,1	5	5	59,67	0	54,232	286930000	17	1,852
*IDH2	Isocitrate dehydrogenase [NADP], mitochondrial	P48735	36,9	36,9	10	10	50,909	0	99,902	1093000000	57	1,852
ARRB1	Beta-arrestin-1	P49407	6,5	6,5	3	3	47,065	0	25,488	312370000	20	2,146
GNAQ	Guanine nucleotide-binding protein G(q) subunit alpha	P50148	10,3	10,3	4	4	42,142	0	58,867	592940000	25	1,852
VASP	Vasodilator-stimulated phosphoprotein	P50552	18,9	18,9	6	6	39,829	0	67,652	3290000000	73	1,023
RAB7A	Ras-related protein Rab-7a	P51149	48,8	48,8	9	9	23,489	0	57,722	774510000	43	1,550
RAB13	Ras-related protein Rab-13	P51153	31	25,6	4	3	22,774	0	42,552	266730000	16	1,991
PGD	6-phosphogluconate dehydrogenase, decarboxylating	P52209	5,4	5,4	3	3	53,139	0	16,249	183850000	8	2,828

ARHGDIB	Rho GDP-dissociation inhibitor 2	P52566	28,4	28,4	3	3	22,988	0	47,748	609320000	33	1,112
CAPZA1	F-actin-capping protein subunit alpha-1	P52907	23,1	19,6	4	3	32,922	0	37,334	511210000	26	1,418
PTTG1IP	Pituitary tumor-transforming gene 1 protein-interacting protein	P53801	18,9	18,9	2	2	20,324	0	70,361	548090000	24	1,133
NAPA	Alpha-soluble NSF attachment protein	P54920	10,5	10,5	2	2	33,232	0	40,686	186940000	12	2,828
VCP	Transitional endoplasmic reticulum ATPase	P55072	27,4	27,4	12	12	89,321	0	169,34	979700000	53	1,852
NAP1L1	Nucleosome assembly protein 1-like 1	P55209	13	13	3	3	45,374	0	21,615	593800000	17	1,852
MTPN	Myotrophin	P58546	36,4	36,4	4	4	12,895	0	54,981	1009300000	54	0,787
TPI1	Triosephosphate isomerase	P60174	44,8	44,8	7	7	30,791	0	224,42	1902700000	95	1,041
MYL6	Myosin light polypeptide 6	P60660	47,7	47,7	4	4	16,93	0	209,4	2393700000	100	0,179
ACTB	Actin, cytoplasmic 1	P60709	73,9	4,5	34	1	41,736	0	323,31	2,252E+11	1411	0,009
CDC42	Cell division control protein 42 homolog	P60953	38,7	33	7	6	21,258	0	64,215	890700000	62	1,852
RAB8A	Ras-related protein Rab-8A	P61006	29	23,7	5	4	23,668	0	108,23	842730000	39	1,852
RAB10	Ras-related protein Rab-10	P61026	37,5	32	6	5	22,541	0	167,84	1563800000	44	1,852
RAB14	Ras-related protein Rab-14	P61106	51,2	51,2	8	8	23,897	0	87,784	507820000	37	2,148
ACTR3	Actin-related protein 3	P61158	31,6	31,6	8	8	47,371	0	143,45	3690000000	91	0,490
ACTR2	Actin-related protein 2	P61160	44,4	44,4	12	12	44,76	0	142,71	1586200000	76	1,562
RAP1B	Ras-related protein Rap-1b	P61224	74,5	36,4	12	4	20,825	0	323,31	8786700000	142	0,390
RAP2B	Ras-related protein Rap-2b	P61225	44,3	44,3	5	5	20,504	0	34,731	286040000	13	2,828
RHOA	Transforming protein RhoA	P61586	23,3	23,3	5	5	21,768	0	71,655	1087900000	71	0,898
HSPE1-MOB4	10 kDa heat shock protein, mitochondrial	S4R3N1	13,8	13,8	2	2	29,736	0	24,375	394110000	28	1,852
B2M	Beta-2-microglobulin	P61769	71,4	71,4	6	6	13,714	0	225,7	4921000000	112	0,441
GNG11	Guanine nucleotide-binding protein G(I)/G(S)/G(O) subunit gamma-11	P61952	35,6	35,6	2	2	8,4807	0	21,497	609480000	25	2,153
YWHAG	14-3-3 protein gamma	P61981	37,7	32,4	9	6	28,302	0	170,59	2140800000	109	0,251
YWHAE	14-3-3 protein epsilon	P62258	37,3	37,3	7	7	29,174	0	117,89	1919100000	90	1,196
TMSB4X	Thymosin beta-4	P62328	84,1	84,1	4	4	5,0526	0	52,184	10489000000	113	0,689
RAB11A	Ras-related protein Rab-11A	P62491	26,4	26,4	5	5	24,393	0	133	2370600000	75	1,146
RAB1A	Ras-related protein Rab-1A	P62820	26,3	21	5	3	22,677	0	52,433	618810000	28	1,242
RAN	GTP-binding nuclear protein Ran	P62826	17,1	17,1	3	3	24,423	0	18,886	153890000	15	1,997
GNB1	Guanine nucleotide-binding protein G(I)/G(S)/G(T) subunit beta-1	P62873	27,6	27,6	4	4	37,377	0	234,28	899800000	32	0,954
PPIA	Peptidyl-prolyl cis-trans isomerase A	P62937	97,6	97,6	17	17	18,012	0	323,31	15969000000	293	0,023
FKBP1A	Peptidyl-prolyl cis-trans isomerase FKBP1A	P62942	32,4	32,4	3	3	11,951	0	34,729	322690000	22	2,828
RAC1	Ras-related C3 botulinum toxin substrate 1	P63000	49,5	30,7	9	6	21,45	0	121,01	2157900000	78	1,852
YWHAZ	14-3-3 protein zeta/delta	P63104	55,5	55,5	16	16	27,745	0	323,31	21058000000	312	0,027
EIF5A	Eukaryotic translation initiation factor 5A-1	P63241	49,4	49,4	5	5	16,832	0	115,86	633820000	41	1,852
ACTG1	Actin, cytoplasmic 2	P63261	73,9	4,5	34	1	41,792	0	164,44	16231000000	74	1,545
TPM4	Tropomyosin alpha-4 chain	P67936	57,7	34,3	14	10	28,521	0	323,31	7515600000	188	0,288
ACTC1	Actin, alpha cardiac muscle 1	P68032	60,2	26,3	26	6	42,019	0	155,18	27357000000	155	0,123
TUBA1B	Tubulin alpha-1B chain	P68363	43	0	12	0	50,151	0	316,28	9209300000	200	0,043
TUBA4A	Tubulin alpha-4A chain	P68366	44,2	11,4	12	3	49,924	0	78,142	1082900000	39	2,828
TUBB4B	Tubulin beta-4B chain	P68371	57,8	16,4	13	3	49,83	0	164,21	828150000	35	1,097
HBB	Hemoglobin subunit beta	P68871	57,8	57,8	7	7	15,998	0	106,97	2505400000	72	1,854
HBA1	Hemoglobin subunit alpha	P69905	75,4	75,4	8	8	15,257	0	223,13	2793900000	93	1,071
GSTO1	Glutathione S-transferase omega-1	P78417	47,3	47,3	8	8	27,566	0	100,6	870800000	41	1,852

DCD	Dermcidin	P81605	33,6	33,6	3	3	11,284	0	36,066	300390000	12	1,983
MPP1	55 kDa erythrocyte membrane protein	Q00013	6	6	2	2	52,296	0	23,076	271200000	17	1,852
CAP1	Adenylyl cyclase-associated protein 1	Q01518	63,6	63,6	24	24	51,901	0	323,31	8098900000	233	0,232
YWHAH	14-3-3 protein eta	Q04917	31,7	26,4	7	5	28,218	0	118,48	1019200000	44	1,425
CALD1	Caldesmon	Q05682	3,9	3,9	2	2	93,23	0	31,462	79699000	10	1,985
ARHGAP1	Rho GTPase-activating protein 1	Q07960	15,5	15,5	4	4	50,435	0	26,932	281120000	15	2,828
CD47	Leukocyte surface antigen CD47	Q08722	7,7	7,7	3	3	35,213	0	55,455	912910000	26	1,897
NEXN	Nexilin	Q0ZGT2	6,2	6,2	3	3	80,657	0	74,204	239490000	18	2,132
PTPRJ	Receptor-type tyrosine-protein phosphatase eta	Q12913	11,3	11,3	9	9	145,94	0	92,131	1753400000	94	1,037
MMRN1	Multimerin-1	Q13201	16,9	16,9	16	16	138,11	0	323,31	4880600000	210	0,613
ILK	Integrin-linked protein kinase	Q13418	47,3	47,3	17	17	51,419	0	323,31	15008000000	354	0,019
COTL1	Coactosin-like protein	Q14019	35,9	35,9	5	5	15,945	0	271,1	4004100000	130	0,027
SEPT11	Septin-11	Q9NVA2	10,7	10,7	3	3	49,398	0	52,242	244980000	17	1,910
RASA3	Ras GTPase-activating protein 3	Q14644	14,9	14,9	9	9	95,698	0	75,869	854840000	49	1,202
LTBP1	Latent-transforming growth factor beta-binding protein 1	Q14766	2,3	2,3	3	3	186,79	0	40,787	748040000	33	1,852
RSU1	Ras suppressor protein 1	Q15404	63,9	63,9	10	10	31,54	0	323,31	4073600000	96	0,470
MAPRE2	Microtubule-associated protein RP/EB family member 2	Q15555	20,8	20,8	6	6	37,031	0	87,718	1234800000	57	1,097
MAPRE1	Microtubule-associated protein RP/EB family member 1	Q15691	25,7	25,7	5	5	29,999	0	110,02	652860000	34	1,852
STXBP2	Syntaxin-binding protein 2	Q15833	39,1	39,1	17	17	66,452	0	228,42	1568400000	97	0,942
ZYX	Zyxin	Q15942	17,3	17,3	5	5	61,277	0	234,29	2544700000	53	0,973
UGP2	UTP--glucose-1-phosphate uridylyltransferase	Q16851	17,1	17,1	6	6	56,94	0	49,297	320750000	26	1,985
CYFIP1	Cytoplasmic FMR1-interacting protein 1	Q7L576	13,6	8,9	15	9	145,18	0	122,73	806710000	51	1,852
FERMT3	Fermitin family homolog 3	Q86UX7	39,6	39,6	21	21	75,952	0	323,31	12073000000	301	0,031
ABI1	Abl interactor 1	Q8IZP0	16,9	16,9	4	4	55,08	0	152,2	454370000	31	1,902
TSPAN14	Tetraspanin-14	Q8NG11	9,3	9,3	2	2	30,69	0	17,919	1003100000	28	2,135
*SLC44A1	Choline transporter-like protein 1	Q8WWI5	6,7	6,7	4	4	73,301	0	39,072	342680000	26	1,852
ATP2A3	Sarcoplasmic/endoplasmic reticulum calcium ATPase 3	Q93084	12,5	7,3	7	4	113,98	0	83,203	735120000	29	0,960
SYTL4	Synaptotagmin-like protein 4	Q96C24	13	13	9	9	76,023	0	61,017	339410000	33	1,852
CNN2	Calponin-2	Q99439	29,4	29,4	5	5	33,697	0	63,979	892600000	57	0,550
*PARK7	Protein deglycase DJ-1	Q99497	38,1	38,1	4	4	19,891	0	44,009	548190000	15	1,902
MGLL	Monoglyceride lipase	Q99685	20,8	20,8	4	4	33,261	0	53,996	252230000	24	1,852
RAB1B	Ras-related protein Rab-1B	Q9H0U4	39,8	34,3	7	5	22,171	0	86,673	3132900000	90	0,133
SH3BGRL3	SH3 domain-binding glutamic acid-rich-like protein 3	Q9H299	32,3	32,3	2	2	10,438	0	40,641	6193100000	58	1,852
TUBB1	Tubulin beta-1 chain	Q9H4B7	65,2	65,2	16	15	50,326	0	323,31	7111000000	181	0,242
EHD1	EH domain-containing protein 1	Q9H4M9	37,3	25,1	15	10	60,626	0	296,07	3310000000	117	1,196
PARVB	Beta-parvin	Q9HBI1	28,6	28,6	10	10	41,714	0	323,31	5131400000	135	1,481
PDLIM7	PDZ and LIM domain protein 7	Q9NR12	11,2	11,2	4	4	49,844	0	34,69	194870000	18	2,129
SAR1A	GTP-binding protein SAR1a	Q9NR31	41,9	41,9	5	5	22,367	0	64,174	405830000	20	1,903
EHD3	EH domain-containing protein 3	Q9NZN3	35,1	23,2	15	10	60,886	0	151,39	1532200000	55	1,458
DBNL	Drebrin-like protein	Q9UJU6	11,2	11,2	3	3	48,207	0	87,811	1117900000	41	1,380
CORO1C	Coronin-1C	Q9ULV4	31,2	31,2	12	12	53,248	0	143,87	2117000000	108	0,495
HPSE	Heparanase	Q9Y251	6,8	6,8	4	4	61,148	0	28,082	304000000	31	1,852

*VDAC3	Voltage-dependent anion-selective channel protein 3	Q9Y277	27,9	27,9	6	6	30,658	0	79,76	585230000	41	1,852
NCKAP1	Nck-associated protein 1	Q9Y2A7	7,5	7,5	7	7	128,79	0	76,591	325350000	19	2,143
TLN1	Talin-1	Q9Y490	63,4	63,4	136	136	269,76	0	323,31	2,0892E+11	2307	0,018
F11R	Junctional adhesion molecule A	Q9Y624	20,1	20,1	4	4	32,583	0	129,47	1886600000	93	0,226
STK24	Serine/threonine-protein kinase 24	Q9Y6E0	14,2	14,2	4	4	49,307	0	28,993	269000000	18	1,852
WASF2	Wiskott-Aldrich syndrome protein family member 2	Q9Y6W5	10,2	10,2	4	4	54,283	0	27,88	287290000	24	1,905

*mitochondrial protein
