# Supplementary material for: Platelet-Derived Microparticles From Obese Individuals: Characterization of Number, Size, Proteomics, and Crosstalk With Cancer and Endothelial Cells
Source: Front Pharmacol. 2019 Jan 22;10:7. doi: 10.3389/fphar.2019.00007 (PMC6349702; doi:10.3389/fphar.2019.00007)
Supplement: Supplementary file 2 [file Table_2.pdf]

Supplementary Table 2

Gene names	Protein names	Protein IDs	Unique Proteins	P value	Fold change	Difference
**CANX**	calnexin	P27824	Unique in healthy			
**PDE5A**	cGMP-specific 3,5-cyclic phosphodiesterase	O76074	Unique in healthy			
**CSK**	Tyrosine-protein kinase CSK	P41240	Unique in Obese			
***GSR**	Glutathione reductase, mitochondrial	P00390	Unique in Obese			
***UBA1**	ubiquitin like modifier activating enzyme 1	P22314	Unique in Obese			
**F5**	Coagulation factor V	P12259		0,0079	3,635	1,862
**PF4V1**	Platelet factor 4 variant	P10720		0,0277	4,938	2,304
**PPBP**	Platelet basic protein	P02775		0,0016	4,102	2,036
**ACTB**	Actin, cytoplasmic 1	P60709		0,0050	0,298	-1,745
**ACTN4**	Alpha-actinin-4	O43707		0,0063	0,294	-1,768
**ACTR3**	Actin-related protein 3	P61158		0,0164	0,209	-2,257
**ADH5**	Alcohol dehydrogenase class-3	P11766		0,0276	0,397	-1,334
**ALDOA**	Fructose-bisphosphate aldolase A	P04075		0,0318	0,294	-1,768
**ARHGDIB**	Rho GDP-dissociation inhibitor 2	P52566		0,0386	0,179	-2,485
**ARPC1B**	Actin-related protein 2/3 complex subunit 1B	O15143		0,0144	0,346	-1,530
**ARRB1**	Beta-arrestin-1	P49407		0,0234	0,411	-1,284
**ATP2A3**	Sarcoplasmic/endoplasmic reticulum calcium ATPase 3	Q93084		0,0324	0,302	-1,728
**B2M**	Beta-2-microglobulin	P61769		0,0184	0,181	-2,464
**CALD1**	Caldesmon	Q05682		0,0155	0,228	-2,133
**CD9**	CD9 antigen	P21926		0,0101	0,269	-1,894
**CFL1**	Cofilin-1	P23528		0,0085	0,398	-1,331
**CNN2**	Calponin-2	Q99439		0,0014	0,173	-2,531
**F11R**	Junctional adhesion molecule A	Q9Y624		0,0059	0,367	-1,445
**FERMT3**	Fermitin family homolog 3	Q86UX7		0,0192	0,213	-2,232
**GAPDH**	Glyceraldehyde-3-phosphate dehydrogenase	P04406		0,0128	0,440	-1,183

***GSTP1**	Glutathione S-transferase P	P09211	0,0316	0,321	-1,642
**HSPA5**	78 kDa glucose-regulated protein	P11021	0,0074	0,318	-1,653
**ITGA2B**	Integrin alpha-IIb	P08514	0,0011	0,225	-2,152
**LDHB**	L-lactate dehydrogenase B chain	P07195	0,0002	0,162	-2,625
**LIMS1**	LIM and senescent cell antigen-like-containing domain protein 1	P48059	0,0034	0,196	-2,350
**MDH1**	Malate dehydrogenase, cytoplasmic	P40925	0,0014	0,194	-2,369
**MYL6**	Myosin light polypeptide 6	P60660	0,0067	0,293	-1,770
**NEXN**	Nexilin	Q0ZGT2	0,0012	0,210	-2,249
**PDIA3**	Protein disulfide-isomerase A3	P30101	0,0010	0,426	-1,231
**PDLIM1**	PDZ and LIM domain protein 1	O00151	0,0314	0,240	-2,058
**PDLIM7**	PDZ and LIM domain protein 7	Q9NR12	0,0162	0,397	-1,334
**PIP4K2A**	Phosphatidylinositol 5-phosphate 4-kinase type-2 alpha	P48426	0,0061	0,321	-1,638
**PKM**	Pyruvate kinase PKM	P14618	0,0146	0,281	-1,834
**PLEK**	Pleckstrin	P08567	0,0060	0,110	-3,185
**PTTG1IP**	Pituitary tumor-transforming gene 1 protein-interacting protein	P53801	0,0149	0,147	-2,768
**RAB13**	Ras-related protein Rab-13	P51153	0,0204	0,373	-1,423
**RAN**	GTP-binding nuclear protein Ran	P62826	0,0106	0,244	-2,037
**RHOA**	Transforming protein RhoA	P61586	0,0223	0,331	-1,594
**SDPR**	Serum deprivation-response protein	O95810	0,0021	0,307	-1,702
**SELP**	P-selectin	P16109	0,0027	0,136	-2,874
**STOM**	Erythrocyte band 7 integral membrane protein	P27105	0,0008	0,102	-3,295
**TAGLN2**	Transgelin-2	P37802	0,0061	0,259	-1,952
**TPM1**	Tropomyosin alpha-1 chain	P09493	0,0026	0,506	-0,983
**TUBB**	Tubulin beta chain	P07437	0,0031	0,212	-2,236

**TUBB4B**	Tubulin beta-4B chain	P68371	0,0290	0,130	-2,945
**TXN**	Thioredoxin	P10599	0,0081	0,432	-1,210

Legend:

Transmembrane, alpha-granule

In red upregulated proteins

In green dowregulated proteins

*mitochondrial proteins
